# Recognizing Student Misconceptions through Ed's Tools and the Biology Concept Inventory

**DOI:** 10.1371/journal.pbio.0060003

**Published:** 2008-01-03

**Authors:** Michael W Klymkowsky, Kathy Garvin-Doxas

## Abstract

Recognizing student misconceptions is critical for effective teaching. Tools that reveal student thinking indicate that misunderstanding randomness interferes with learning of molecular dynamics and evolutionary processes.

Over the past decade, workers in physics education research have developed effective instructional methods and materials (e.g., workshop physics [1]; lecture demonstrations [2]; tutorials in introductory physics [3]) based on research into student thinking. A Socratic process of questioning and careful analysis of responses can reveal students' thinking on a subject area, including misconceptions, prior conceptions, and conceptual lacunae. Applying this approach to biological concepts, we have built a software system, called Ed's Tools, to capture and analyze student responses. Both instructors and researchers can use this system to obtain a more complete and nuanced picture of student understanding, which can then serve as the foundation on which to base subsequent instruction and the construction of concept inventories. We illustrate the value of the data obtained through this analysis by showing how it helped us trace the conceptual problems that students have in two subject areas, molecular biology and evolutionary biology, to a common cause: a fundamental misunderstanding of random processes.

Random events underlie a wide range of biological processes as diverse as genetic drift and molecular diffusion. Moreover, random events often lead to emergent behaviors, and are exploited in various contexts, from the generation of morphogen gradients during embryonic development [4] to genome evolution [5]. It is also clear that consideration of random events is important to understanding the regulation of gene expression [6–10], cellular differentiation [11], neural network function [12–14], immune system regulation, genome and gene structure [15,16], and presumably homeostatic and adaptive mechanisms in general [17,18].

The importance of random processes in biological systems leads to the obvious question, how well are these concepts currently taught—and understood? The academic study of understanding of randomness among students and the public has a long history, beginning with Piaget and Inhelder ([19]; see also Lecoutre et al., 2006 [20], and references therein). It is well known that understanding the nature and significance of random processes in either abstract scientific systems or “real life” is not easy. Taleb [21] discusses this topic as it applies to our social, financial, and personal experiences and how we interpret their meaning. Lecoutre et al. [20] describe an enlightening study of concepts of randomness and probability among French middle school students, psychology graduate students, and mathematicians, concluding that there exists a significant level of confusion in all groups. Both Lander [4] and Lynch [5] suggest that many professional biologists have problems appreciating the power of random processes.

## Concept Inventories Capture Student Thinking

One way to probe student understanding of random processes in biological systems is through the use of research-based conceptual assessment instruments, such as concept inventories (Box 1). Concept inventories are designed to circumvent various test-taking strategies by using students' own language and misconceptions. The Force Concept Inventory, developed by Hestenes and colleagues [22], focused on student understanding of Newton's laws of motion. The rather disappointing level of understanding evidenced by students taught through standard lecture methods [23] helped trigger the current reform movement in physics education.

Odom and colleagues [24] developed an instrument that examined student understanding of diffusion and osmosis, processes that depend upon the random movement of molecules, while the Biology Concept Inventory or BCI (Box 1; http://bioliteracy.net/) contains a number of questions specifically targeted to conceptual areas in which randomness plays a role [25].

The first step in developing the questions related to randomness on the BCI was collecting data from open-ended questions posed to students at a number of colleges and universities around the country. These included questions such as:

- Describe the mechanism through which the plasma membrane poses a barrier to the movement of hydrophilic molecules.
- Cells and their components can be described as molecular machines. How is the activity of these machines controlled?
- Random events such as genetic drift, founder effects, and bottlenecks can influence evolutionary change in a population. How does this work, and can these processes produce traits that are not adaptive?
- There are a number of processes that influence evolution but which do not act by selecting advantageous traits; how do they work?
- Describe the role of random events in evolutionary processes.
- What is diffusion and why does it occur?
- Imagine that you are a molecule of ADP inside a cell. Describe how you manage to “find” an ATP synthase, so that you can become an ATP.

Student responses to these questions, together with student interviews, form the basis for the construction and validation of BCI questions [25] (Box 1).

Results from the BCI indicate a striking lack of understanding on two questions related to randomness, even after three major's courses in Molecular, Cell, and Developmental Biology at the University of Colorado at Boulder—we suspect that similar results would be found widely. BCI results were strongly supported (and extended) through “think-aloud” interviews conducted with students. This has led us to consider the broader ramifications of misunderstanding random processes in both students and the general population.

A common observation, which echoes the finding of Lecoutre et al., was that students were unwilling to see random processes as capable of directed effect in themselves—they routinely seek alternative rational explanations, the dominant one being the presumption of drivers that are actually responsible for the observed effects. In the absence of these drivers, for example, concentration gradients with respect to diffusion or active selection with respect to changes in allele frequency, the macroscopic behavior stops. The concept of random processes giving rise to emergent behavior is almost totally absent from their (explicit) thought processes.

**Box 1. What Is a Concept Inventory and How Are They Built?**

**Figure:**
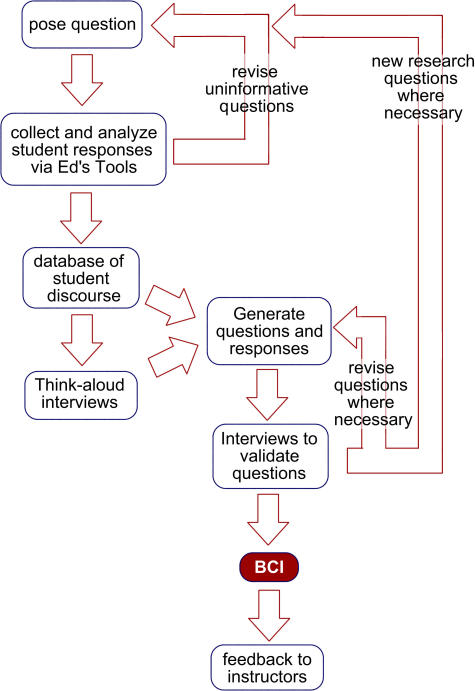

Concept inventories are multiple-choice instruments that explore students' conceptual understanding in a given subject area, providing researchers with a map of their students' conceptual landscape, which can be used to inform instruction in that area. Although concept inventories bear a strong resemblance to standardized tests, their intended use differs from that of tests in crucial ways, which results in significant differences between the way concept inventories and standardized tests are constructed.

Unlike standardized tests, which are typically designed to rank students with respect to subject area knowledge, concept inventories are designed to answer the question “What is the probability that a particular student is using a particular conceptual model when working on a problems in this field?” Concept inventories achieve this goal by using distracters (“wrong” answers) that represent common student misconceptions. Furthermore, they are worded in the language that the students use to express these concepts, not the language that the experts would use. This avoids cluing the students in as to the “correct” answer. Validation of the instrument involves administration of the instrument followed by in-depth interviews of students, with the aim of establishing the percentage of misses and false positives (a miss is a case in which a student holds a particular misconception that the instrument failed to spot, and a false positive is a case in which the instrument spotted a misconception that the student does not actually hold).

In a similar vein, Lynch [5] points out the dichotomy between commonly held misconceptions and uncritically accepted assumptions, and various realities established by evolutionary biologists. For example, #8 on his list (myth): “Phenotypic and genetic modularity are direct products of natural selection” versus (reality): “There is no evidence that the modular structure of gene regulatory regions or genetic networks is directly advanced by selective mechanisms. However, the processes of duplication, degenerative mutation, and random genetic drift can lead to the *passive emergence* [our emphasis] of modularity in populations of…genetic effective sizes of the magnitude found in multicellular species.”

Given that much of evolutionary change is ultimately driven by, or is the result of, random processes rather than selection acting alone (see [5] and references therein), and given the apparent tendency of students to reject or overlook random events as the cause of emergent behaviors, what emerges is “neo-vitalist” mindset that presumes the presence of directed processes and imposes a level of meaning on the system (and its components) that may well not be present. Not all genetic changes have an immediate adaptive significance, and not all molecular processes are actively directed. Does this view interfere with understanding? The answer must be yes—since it leads one to assign purpose to a process (be it evolution or osmosis), and ignores what can be achieved at the underlying molecular level. From an evolutionary perspective, it leads to “just-so” stories that project meaning onto every variation, whether meaningful or not, and obscures the basic mechanisms that make evolutionary theory so valuable. On the molecular biology level it leads to anthropomorphic explanations of molecular interactions, some of which even imply action at a distance; e.g., ATP synthase molecules “seek out and grab” ADP molecules [25].

**Box 2. Using Ed's Tools**

**Figure:**
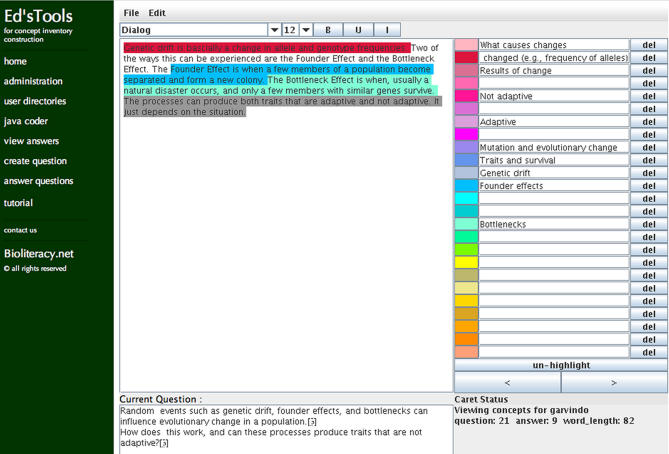

For the development of the BCI, we used Ed's Tools [27] to collect and tag the relevant student language—it is this language that was used to construct the responses. We first assign open-ended questions on the wide subject area (e.g., how is genetic information stored in an organism and how is it used?). We then tag the exact words and phrases that students use in responding to that question, aggregate them, and sort them into concept categories. When constructing the questions and responses, we use as much as possible the words and phrases that the students used. We validate the questions and responses by administering the instrument and then holding in-depth interviews (essentially oral exams) to ascertain the percentage of misses and false positives. Ed's Tools is currently being used in a range of areas (biotechnology, chemistry, physics, computer science) to capture student thinking and language. It is freely available to qualified researchers for their work. All we ask is that we be allowed access to student responses.

## Teaching an Appreciation of Randomness

What, if anything, can be done to improve understanding of the role of random processes in particular, and emergent behaviors in general? Here are some hints. From the perspective of course and curriculum content, we need to provide students with opportunities to work with random systems, and explicitly state (and confront) their assumptions. One approach is through direct experimentation and accessible simulations that focus on the concept of randomness in specific processes. Under these conditions, events like allele loss from a population can be viewed as either the result of selection or genetic drift (or both)—leading to an understanding of the effects of population size on evolutionary effects. In the context of cell biology, students need to directly and explicitly consider the efficacy of diffusion in cellular and organismic context—why is it (apparently) adequate within a bacterial cell, but inadequate for a neuron? For a particular context, one might require students to predict, explaining their thinking, when a process is likely to be active (energy requiring) or passive (diffusive). How can the diffusive properties of a molecule be regulated by intracellular/extracellular structures and molecular interactions? For example, one could ask in which developmental or organismic contexts we can expect diffusion to be adequate (consider [4]). The development of experimental scenarios and/or computer-based simulations can then be used to test and re-evaluate students' assumptions.

Similarly, in the area of gene regulation, it is possible to present scenarios in which fluctuations in RNA and protein levels lead to emergent behavior. In this case, one can ask how the presence of noise is exploited or dealt with in terms of molecular interactions, e.g., as described by Maamar et al. [11]. Again, applets (i.e., student-manipulatable, model-driven computer simulations) can be used as the basis of exercises to test assumptions and to drive students toward a more sophisticated understanding of underlying, and previously unappreciated, processes. (Good examples are the PhET [physics education technology] simulations developed by Carl Weiman's group—see http://phet.colorado.edu/.) Clearly, such an understanding is fundamental to much of modern systems biology.

These are issues that need to be addressed in order to improve the robustness of students' conceptual understanding. In terms of curricular changes, it seems likely that students will need to be provided with the tools, and the contexts in which to use those tools, for dealing with complex processes, including diffusion, drift, and nonlinearity. These are topics that appear to be missing from most biological curricula. Given the constraints on curricula, it is clear that we need to re-examine the conventional curriculum and course content to “make room” for these important topics. If we ignore them, we leave our students open to deep confusion, a particularly pernicious fate for those students who choose to pursue a career in teaching.

## How Ed's Tools Can Help

The results gained from exploring student understanding can lead to dramatic changes in teaching emphasis and methods. While Ed's Tools was developed as a research instrument to facilitate concept inventory development and to assess student understanding, it is also available for instructors to use. For example, an instructor could use one of the existing questions, or pose their own, and ask their students to answer it. They can then use Ed's tools to capture student language related to specific conceptual misunderstandings and lacunae. Student language can provide a jumping-off point for in-class questions (e.g., using “clickers,” or handheld electronic devices, to transmit responses [26]) and discussions, encouraging students to explicitly consider their assumptions as they approach a particular question or problem. Such a multistep analysis of student responses can be key in dramatically improving one's appreciation of how students respond to instructional efforts. You can learn more about Ed's Tools at the Bioliteracy Project home page, http://bioliteracy.net/.

## Abbreviations

BCI: Biology Concept Inventory
